# Supplementary material for: Minimizing non-radiative decay in molecular aggregates through control of excitonic coupling
Source: Nat Commun. 2023 Aug 19;14:5056. doi: 10.1038/s41467-023-40716-w (PMC10439946; doi:10.1038/s41467-023-40716-w)
Supplement: Supplementary file 1 — Supplementary Information [file 41467_2023_40716_MOESM1_ESM.pdf]

Supplementary Information: Minimizing non-radiative decay in molecular aggregates through control of excitonic coupling

Yuanheng Wang,[1] Jiajun Ren,[*,2] and Zhigang Shuai[*,1,3]

1 *MOE Key Laboratory of Organic OptoElectronics and Molecular Engineering, Department of Chemistry, Tsinghua University, Beijing 100084, People's Republic of China*

2 *Key Laboratory of Theoretical and Computational Photochemistry, Ministry of Education, College of Chemistry, Beijing Normal University, Beijing 100875, People's Republic of China*

3 *School of Science and Engineering, The Chinese University of Hong Kong, Shenzhen 518172, People's Republic of China*

E-mail: jjren@bnu.edu.cn; zgshuai@tsinghua.edu.cn

Supplementary Note 1. Influence of the sign of excitonic couplings

1.1 Numerical simulations

In this section, we present our simulations on a dimer model where modes with significant nonadiabatic couplings (promoting modes, PM) have the same electron-phonon couplings as modes with significant electron-phonon couplings (accepting modes, AM), to compare the influence of the sign of excitonic coupling (The Huang-Rhys factors are $S_{\mathrm{PM}} = S_{\mathrm{AM}} = 2$). For

J-aggregates, $J < 0$, and for H-aggregates, $J > 0$. We also plot results from the dimer model used in the main text ($S_{\mathrm{PM}} = 0$ $S_{\mathrm{AM}} = 2$) for comparison. As shown in Supplementary FIG. 1, our results demonstrate that the non-radiative decay rate in J-aggregates and H-aggregates can differ only when the promoting modes with significant nonadiabatic coupling also have significant electron-phonon coupling. This finding is consistent with the discussion in the main text. However, in real-world molecular aggregates, PMs with large nonadiabatic coupling often have small electron-phonon coupling, as demonstrated in the azulene molecule in the main text.

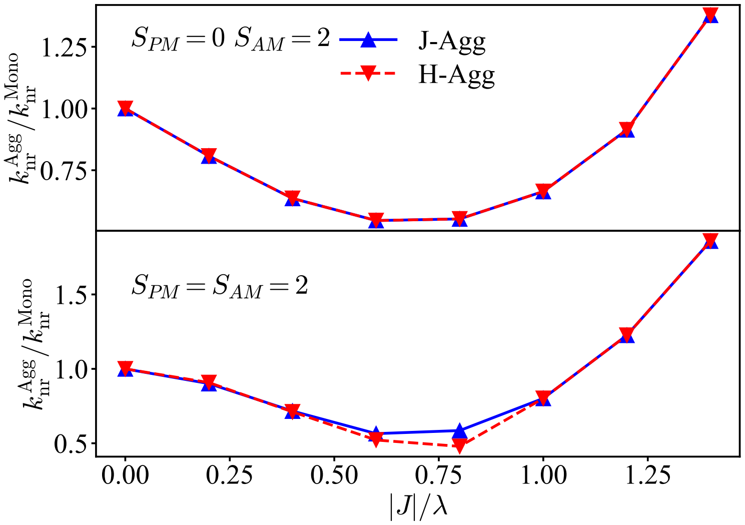

Supplementary Figure 1: Comparisons between the non-radiative rate in J-aggregate (J-Agg) and H-aggregate (H-Agg).

The $k_{\mathrm{nr}}^{\mathrm{Agg}}/k_{\mathrm{nr}}^{\mathrm{Mono}}$ is simulated for both J/H-Agg and when PMs have significant electron-phonon coupling or not.(Promoting modes: PMs; Accepting modes: AMs. S_{PM}andS_{AM} is the Huang-Rhys factors for each kind of modes.)

1.2 Analytical derivations

In order to study the influence of the sign of excitonic coupling analytically, first we need to figure out the difference between the initial states for non-radiative decay in aggregates whose excitonic couplings have the same strength but different signs. The system Hamiltonian can

be expressed as:

$$\hat{H}_0 = \sum_i E_{\mathrm{ad}} a_i^\dagger a_i + \sum_{i,j} J_{i,j} a_i^\dagger a_j + \sum_{i,n} \omega_{i,n}(b_{i,n}^\dagger b_{i,n} + \frac{1}{2}) + \sum_{i,n} g_{i,n}\omega_{i,n} a_i^\dagger a_i (b_{i,n}^\dagger + b_{i,n}) + \sum_{i,n} \lambda_{i,n} a_i^\dagger a_i \tag{1}$$

where i is the index of monomer. E_{ad} is the adiabatic excitation energy between the ground state and the local excited state, which is considered the same for excitation on each monomer. $J_{i,j}$ is the excitonic coupling strength between the local excited states. $a_i^\dagger$ and a_i correspond to the electronic creation/annihilation operators. n indicates the index of vibrational mode on each monomer i. $\omega_{i,n}$ is its harmonic vibrational frequency, g_{in} is its dimensionless electron-phonon coupling strength and $\lambda = g^2\omega$ is its reorganization energy. $b_{i,n}^\dagger$ and $b_{i,n}$ correspond to vibrational creation/annihilation operators. At zero temperature, the initial state $|\Psi_{\mathrm{i}}\rangle$ in the two-mode model dimer studied in the main text is the lowest-energy eigenstate in the one-exciton subspace of corresponding $\hat{H}_0$. Since PM vibrations are not coupled with the rest of degrees of freedoms (Dofs) in $\hat{H}_0$, we can describe $|\Psi_{\mathrm{i}}\rangle = |\widetilde{\Psi}_{\mathrm{i}}\rangle \otimes |\phi_{(0,0)}^{\mathrm{PM}}\rangle$, where $|\phi_{(0,0)}^{\mathrm{PM}}\rangle$ is the lowest-energy eigenstate for PMs and $|\widetilde{\Psi}_{\mathrm{i}}\rangle$ is the lowest-energy eigenstate for the rest of system.

The rest of the system is made up of DoFs of electron and AMs. They are entangled through electron-phonon coupling. So a reasonable eigenstate $|\widetilde{\Psi}\rangle$ should be expanded upon the direct product basis space as Eq. 2. Basis set $\{|\phi_{\mathbf{v}}^{\mathrm{AM}}\rangle = |\phi_{v_1}^{\mathrm{AM}_1}\rangle \otimes |\phi_{v_2}^{\mathrm{AM}_2}\rangle\}$ is used for two AMs and basis set $\{|01\rangle, |10\rangle\}$ is used to describe the electronic states in the one exciton subspace. ($\mathbf{v} = (v_1, v_2)$)

$$|\widetilde{\Psi}\rangle = \sum_{\mathbf{v}}^{d^2} C_{\mathbf{v}}^{|01\rangle}|01\rangle \otimes |\phi_{\mathbf{v}}^{\mathrm{AM}}\rangle + C_{\mathbf{v}}^{|10\rangle}|10\rangle \otimes |\phi_{\mathbf{v}}^{\mathrm{AM}}\rangle \tag{2}$$

The coefficient vector $\mathbf{C} = [\mathbf{C}^{|10\rangle}, \mathbf{C}^{|10\rangle}]$ with length as $2d^2$ can be obtained by diagonalizing a subsystem Hamiltonian $\tilde{\hat{H}}_0$. (d is the basis size used for each mode.) $\tilde{\hat{H}}_0$ includes only terms related to DoFs of electron and AMs in $\hat{H}_0$. The matrix representation of $\tilde{\hat{H}}_0$ can be

obtained by expanding $\widetilde{\hat{H}}_0$ upon only the electronic basis as :

$$\widetilde{\hat{H}}_0 = \langle 01|\widetilde{\hat{H}}_0|01\rangle|01\rangle\langle 01| + \langle 10|\widetilde{\hat{H}}_0|10\rangle|10\rangle\langle 10| + \langle 01|\widetilde{\hat{H}}_0|10\rangle|01\rangle\langle 10| + \langle 10|\widetilde{\hat{H}}_0|01\rangle|10\rangle\langle 01| \quad (3)$$

Using $\hat{H}_0$ represented in Eq. 1, we can find the first two terms in Eq. 3 are diagonal terms that are independent of excitonic coupling J. The latter two terms are off-diagonal terms and share the same value when further expanded on the AMs' basis:

$$\langle 01|\widetilde{\hat{H}}_0|10\rangle = \langle 10|\widetilde{\hat{H}}_0|01\rangle = \sum_{\mathbf{v}',\mathbf{v}} J\langle\phi^{\mathrm{AM}}_{\mathbf{v}'}|\phi^{\mathrm{AM}}_{\mathbf{v}}\rangle|\phi^{\mathrm{AM}}_{\mathbf{v}'}\rangle\langle\phi^{\mathrm{AM}}_{\mathbf{v}}| = \sum_{\mathbf{v}} J|\phi^{\mathrm{AM}}_{\mathbf{v}}\rangle\langle\phi^{\mathrm{AM}}_{\mathbf{v}}| \quad (4)$$

So, the matrix representation of $\widetilde{\hat{H}}_0$ describing two dimer models whose J have the same absolute value but opposite sign would have the same diagonal terms. Their off-diagonal terms would have the same absolute value but opposite sign as shown in Eq. 4. By diagonalizing $\widetilde{\hat{H}}_0$, we can obtain two sets of eigenstates. An one-on-one pair relationship exists between elements in two sets. Within each pair, the eigenstates for the two systems share the same eigenenergy and their coefficients are $\mathbf{C} = [\mathbf{C}^{|01\rangle}, \mathbf{C}^{|10\rangle}]$ and $\mathbf{C}' = [\mathbf{C}^{|01\rangle}, -\mathbf{C}^{|10\rangle}]$. So we can write a pair of initial states at zero temperature (lowest energy eigenstates) in two dimer systems with the same excitonic couplings strength but different signs as:

$$|\Psi^{J=J_0}_{\mathrm{i}}\rangle = (|01\rangle \otimes |\varphi^{\mathrm{AM}}_{|01\rangle}\rangle + |10\rangle \otimes |\varphi^{\mathrm{AM}}_{|10\rangle}\rangle) \otimes |\phi^{\mathrm{PM}}_{(0,0)}\rangle \quad (5)$$

$$|\Psi^{J=-J_0}_{\mathrm{i}}\rangle = (|01\rangle \otimes |\varphi^{\mathrm{AM}}_{|01\rangle}\rangle - |10\rangle \otimes |\varphi^{\mathrm{AM}}_{|10\rangle}\rangle) \otimes |\phi^{\mathrm{PM}}_{(0,0)}\rangle \quad (6)$$

$$|\varphi^{\mathrm{AM}}_{|01\rangle/|10\rangle}\rangle = \sum_{\mathbf{v}}^{d^2} C^{|01\rangle/|10\rangle}_{\mathrm{i},\mathbf{v}}|\phi^{\mathrm{AM}}_{\mathbf{v}}\rangle \quad (7)$$

The two initial states share the same energy E_{i}. The specific value of coefficient vector $\mathbf{C}_{\mathrm{i}} = [\mathbf{C}^{|01\rangle}_{\mathrm{i}}, \mathbf{C}^{|10\rangle}_{\mathrm{i}}]$ for the initial state can be obtained through diagonalization or other ground state optimization algorithm like DMRG. However, as its specific value is not important for discussion here, we don't need to calculate it. The possible final states for non-radiative decay

should be in the zero-exciton space. In zero-exciton space, all Dofs in $\hat{H}_0$ are independent with each other. So final states can be expressed as:

$$|\Psi_{\rm f}\rangle = |00\rangle \otimes |\phi^{\rm AM}_{\mathbf{v}^{\rm AM}}\rangle \otimes |\phi^{\rm PM}_{\mathbf{v}^{\rm PM}}\rangle \tag{8}$$

The nonadiabatic coupling term in this two-mode model dimer is

$$\hat{H}_1 = \big(\langle 00|\hat{p}_1|01\rangle|00\rangle\langle 01| + \text{h.c.}\big)\hat{p}_1 + \big(\langle 00|\hat{p}_2|10\rangle|00\rangle\langle 10| + \text{h.c.}\big)\hat{p}_2 \tag{9}$$

$$= \big(V|00\rangle\langle 01| + \text{h.c.}\big)\hat{p}_1 + \big(V|00\rangle\langle 10| + \text{h.c.}\big)\hat{p}_2 \tag{10}$$

where $V = \langle 00|\hat{p}_1|01\rangle = \langle 00|\hat{p}_2|10\rangle$. $\hat{p}_1, \hat{p}_2$ are momentum operators of PM on each of the two monomers. For harmonic PMs, we have

$$\langle\phi^{\rm PM}_{(v_2,v_1)}|\hat{p}_1|\phi^{\rm PM}_{(0,0)}\rangle = \delta_{v_2,0}\delta_{v_1,1}\zeta \tag{11}$$

$$\langle\phi^{\rm PM}_{(v_2,v_1)}|\hat{p}_2|\phi^{\rm PM}_{(0,0)}\rangle = \delta_{v_2,1}\delta_{v_1,0}\zeta \tag{12}$$

where ζ is also a constant whose specific value is not important for discussions here. Then the matrix elements among the summation in Fermi's Golden Rule (FGR, Eq.5 in the main text) should be nonzero only when: $|\Psi_{\rm f}\rangle = |00\rangle \otimes |\phi^{\rm AM}_{\mathbf{v}^{\rm AM}}\rangle \otimes |\phi^{\rm PM}_{(0,1)}\rangle$ or $|\Psi_{\rm f}\rangle = |00\rangle \otimes |\phi^{\rm AM}_{\mathbf{v}^{\rm AM}}\rangle \otimes |\phi^{\rm PM}_{(1,0)}\rangle$. They also share a series of the same energy $E_{{\rm f},\mathbf{v}^{\rm AM}}$. Finally, we obtain the same $k_{\rm nr}$ expression as in Eq. 13 for both of the two initial state $|\Psi_{\rm i}^{J=J_0}\rangle$ and $|\Psi_{\rm i}^{J=-J_0}\rangle$. The same expression explains the same non-radiative decay behavior simulated in the main text for aggregates whose excitonic couplings have the same strength but opposite signs.

$$k_{\rm nr} = \sum_{\mathbf{v}^{\rm AM}} (|V\zeta\langle\phi^{\rm AM}_{\mathbf{v}^{\rm AM}}|\varphi^{\rm AM}_{|01\rangle}\rangle|^2 + |V\zeta\langle\phi^{\rm AM}_{\mathbf{v}^{\rm AM}}|\varphi^{\rm AM}_{|10\rangle}\rangle|^2)\delta(E_{{\rm f},\mathbf{v}^{\rm AM}} - E_{\rm i}) \tag{13}$$

Supplementary Note 2. Computational setup for TD-DMRG simulations

2.1 Dimer model

For the zero temperature simulation in the dimer model, the bond dimension $M = 10$ is used for initial state optimization and real-time evolution. For the finite temperature simulation in the dimer model, the bond dimension $M = 10$ is used for the imaginary-time evolution to obtain the initial thermal equilibrium state and the following real-time evolution to obtain the time correlation functions. In both cases, the simple harmonic oscillator (SHO) basis with basis size $d = 30$ is used for each mode. (When studying the influence of low frequency torsion modes in the main text, the basis size used for low-frequency modes is also $d = 30$ for the simulation at zero temperature.) The frequency of SHO basis is set to be the same as the vibrational frequency ω for corresponding vibrational modes on the electronic ground state. The centers of SHO basis are located at the equilibrium point of the electronic ground state. Time step $\mathrm{d}t = 0.2\,\mathrm{fs}$ is used to perform the real-time evolution. Altogether 100 steps are propagated.

2.2 1D chain model

For the zero temperature simulation, $M = 10$ is used for initial state optimization and real-time evolution. For finite temperature simulation, $M = 30$ is used for the imaginary-time evolution to obtain the initial thermal equilibrium state and the following real-time evolution to obtain the time correlation functions. In both situations, the basis used for each vibration is the same as in the dimer model. For simulations at room temperature with inter-molecular vibrations between each nearest-neighbor sites, $M = 30$ is used for the imaginary-time evolution to obtain the initial thermal equilibrium state and the following real-time evolution to obtain the time correlation functions. While SHO basis with basis size

$d = 30$ is used for intra-molecular vibrations, $d_{\text{inter}} = 40$ is used for inter-molecular vibrations. Also, time step $\mathrm{d}t = 0.2\,\mathrm{fs}$ is used to perform the real-time evolution. Altogether 100 steps are propagated.

2.3 2D square lattice model

For the zero temperature simulation, $M = 25$ is used for initial state optimization and also $M = 10$ is used for the real-time evolution. For finite temperature simulation, $M = 50$ is used for the imaginary-time evolution to obtain the initial thermal equilibrium state and the following real-time evolution to obtain the time correlation functions for $|J|/\lambda = 0 \sim 0.3$. $M = 70$ is used for $|J|/\lambda = 0.4 \sim 0.6$ and $M = 80$ is used for $|J|/\lambda = 0.7$ cases to deal with the strong long-range couplings artificially introduced when mapping 2D square lattice to 1D matrix product state ansatz. In both situations, the basis used for each vibration is the same as that in the dimer model. Also, the time step $\mathrm{d}t = 0.2\,\mathrm{fs}$ is used to perform the real-time evolution. Altogether 100 steps are propagated.

2.4 Azulene dimer

Only zero temperature simulations are performed for the azulene dimer, the bond dimension $M = 10$ is used for initial state optimization and real-time evolution. The simple harmonic oscillator (SHO) basis with basis size $d = 10$ is used for each mode. The frequency of SHO basis is set to be the same as the vibrational frequency ω for corresponding vibrational modes on the electronic ground state. The centers of SHO basis are located at the equilibrium point of the electronic ground state. Time step $\mathrm{d}t = 0.1\,\mathrm{fs}$ is used to perform the real-time evolution. Altogether 2000 steps are propagated.

2.5 Broadening functions

A Gaussian form broadening function $g(t) = e^{-\sigma^2 t^2/2}$ with $\sigma = 0.008\,\text{a.u.}$ is applied to the time correlation functions (TCFs) in aggregates of two-mode simplified model cases and $\sigma = 0.0008\,\text{a.u.}$ in azulene dimer case to ensure that the TCFs decay to zero within the simulated finite time-scale. Then we can only perform time integration in this finite time to calculate the required nonradiative rates. Supplementary FIG. 2 is a typical demonstration of TCFs in this work before and after applying a Gaussian form broadening function.

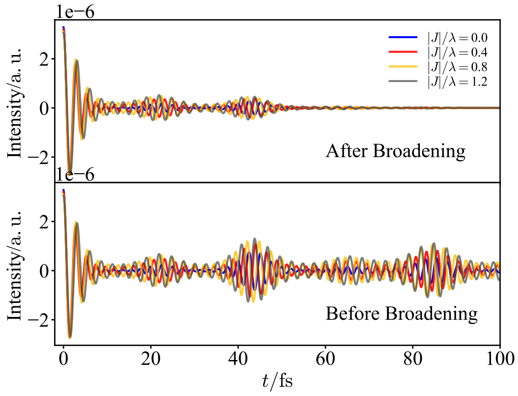

Supplementary Figure 2: The time correlation function before and after applying a broadening function.

The time correlation functions for non-radiative decay of azulene dimer with different excitonic coupling strength $|J|$ at zero temperature before (bottom panel) and after (upper panel) applying a Gaussian form broadening function. (The monomer reorganization energy λ is used as the unit here)

2.6 Ordering of MPS

For the dimer model and 1D chain model, the electronic DoFs are mapped to an MPS chain according to their real space geometry. In the 2D square lattice model, we arrange the electronic DoFs row by row. The electronic DoFs in each row are mapped into the MPS chain from left to right. The leftmost electronic DoF is linked to the rightmost electronic DoF of the previous row. The vibrational DoFs for each electronic DoF are placed next to the electronic DoF.

2.7 Convergence benchmarks for TD-DMRG simulations

Here, we benchmark the computational parameters used for TD-DMRG simulations in the main text to confirm the results are numerically converged. The Huang-Rhys factor $S = 0.5$ is used for all benchmarks here and the physical parameters are the same as the corresponding system in the main text.

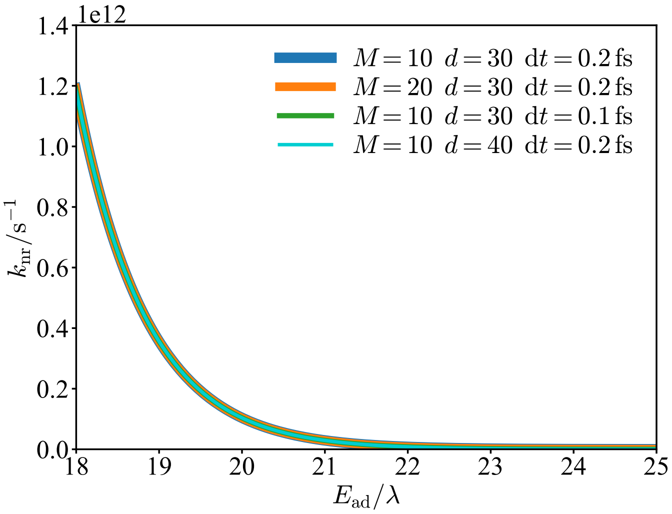

Supplementary Figure 3: Non-radiative decay rate (k_{nr}) spectrum results for dimer at zero temperature when $|J|/\lambda = 1.4$ from different time-dependent density matrix renormalization group (TD-DMRG) computational parameters including the bond dimension M; basis size d; time-step size for real-time evolution $\mathrm{d}t$.

It can be seen all computational parameters used for simulations in the main text are already converged.(E_{ad} is the adiabatic excitation energy for each monomer)

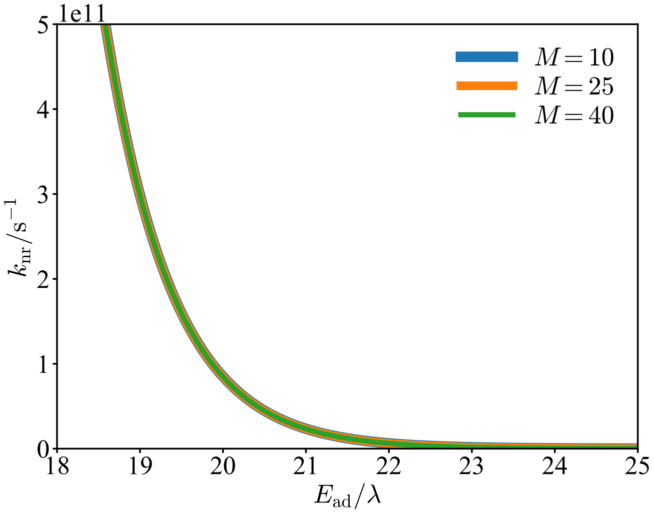

Supplementary Figure 4: Non-radiative decay rate (k_{nr}) spectrum results for 1D chain at zero temperature when $|J|/\lambda = 1.0$ simulated by time-dependent density matrix renormalization group (TD-DMRG) method with different bond dimension M.

The result from $M = 10$ has been converged to that of larger M. So $M = 10$ is used in the main text simulations.(E_{ad} is the adiabatic excitation energy for each monomer)

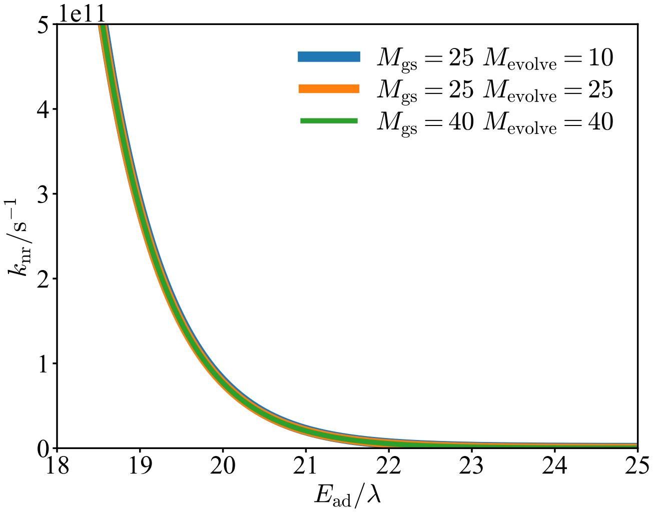

Supplementary Figure 5: Nonradiative decay rate (k_{nr}) spectrum results for 2D square lattice at zero temperature when $|J|/\lambda = 0.7$ from different bond dimensions M. In this case, we use different M (M_{gs} and M_{evolve}) for the density matrix renormalization group (DMRG) ground state optimization algorithm (Here we use it to obtain the lowest energy eigenstate within one-exciton subspace) and real-time evolution when performing time-dependent density matrix renormalization group (TD-DMRG) simulation in the main text.

The result from $M_{gs} = 25$ and $M_{evolve} = 10$ has been converged to that of larger M. So they are used in the main text simulations.(E_{ad} is the adiabatic excitation energy for each monomer)

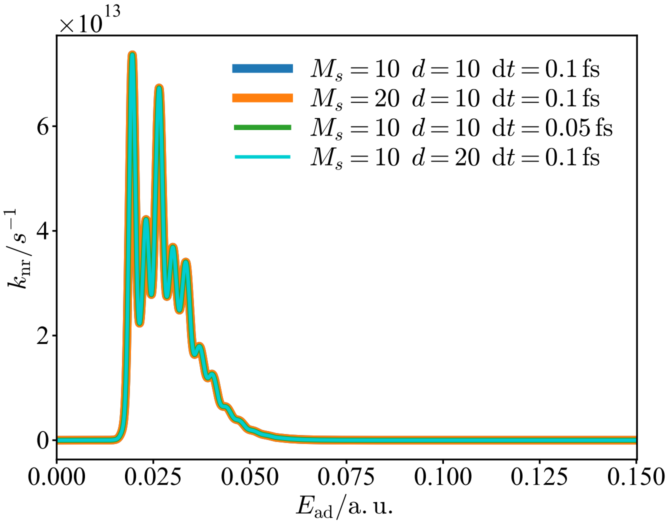

Supplementary Figure 6: Non-radiative decay rate (k_{nr}) spectrum results for the azulene dimer at zero temperature when $|J|/\lambda = 1.2$ from different time-dependent density matrix renormalization group (TD-DMRG) computational parameters including the bond dimension M; basis size d; time-step size for real-time evolution dt.

Unlike in the two-mode simplified model aggregates cases, the full spectrum is calculated here because it is also discussed in the main text. It can be seen all computational parameters used for simulations in the main text are already converged.(E_{ad} is the adiabatic excitation energy for each monomer)

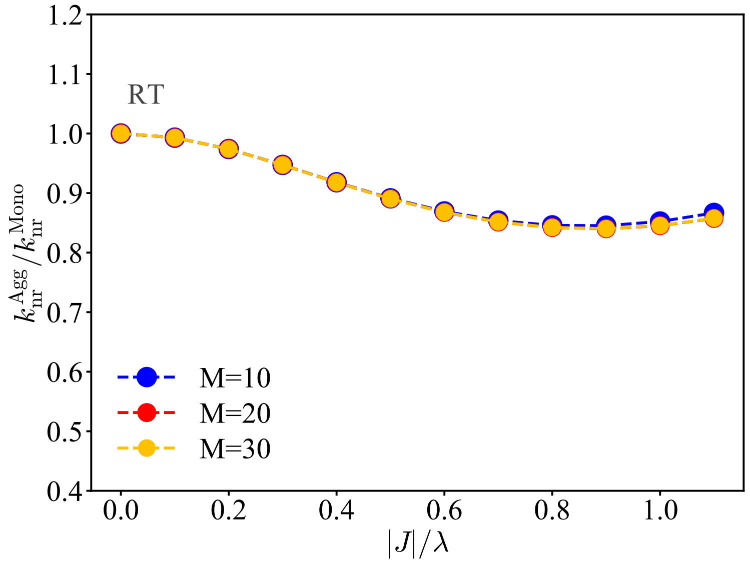

Supplementary Figure 7: Benchmark non-radiative decay rate $k_{\mathrm{nr}}^{\mathrm{Agg}}/k_{\mathrm{nr}}^{\mathrm{Mono}}$ results for different bond dimension M used in TD-DMRG simulation in dimer at room temperature (RT).

The result has already been converged with $M = 10$. So it is used in the main text.

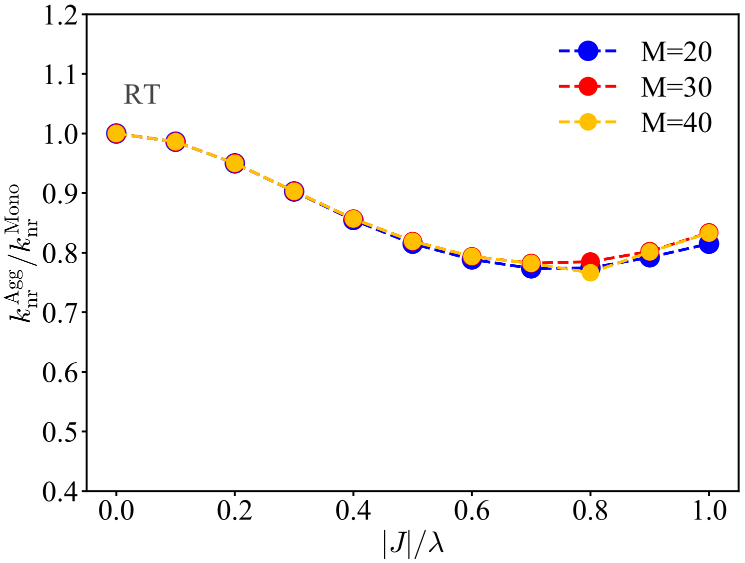

Supplementary Figure 8: Benchmark non-radiative decay rate $k_{\rm nr}^{\rm Agg}/k_{\rm nr}^{\rm Mono}$ results for different bond dimension M used in time-dependent density matrix renormalization group (TD-DMRG) simulation in 1D chain at room temperature (RT).

The result is converged with $M = 30$. So it is used in the main text. The bond dimension is smaller than that used in the 2D square lattice.

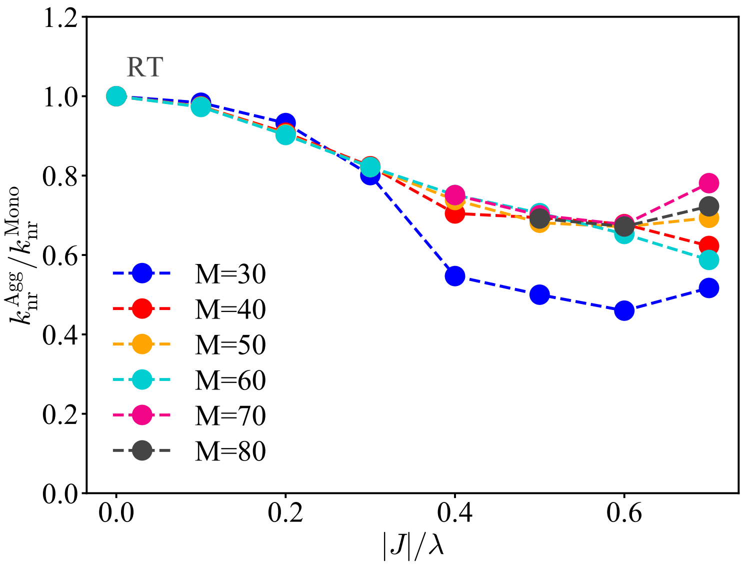

Supplementary Figure 9: Benchmark non-radiative decay rate $k_{\rm nr}^{\rm Agg}/k_{\rm nr}^{\rm Mono}$ results for different bond dimension M used in time-dependent density matrix renormalization group (TD-DMRG) simulation in 2D square lattice at room temperature (RT).

The $M = 50$ result is qualitatively right compared to that of larger M when $|J|/\lambda < 0.4$. So we use $M = 50$ for $|J|/\lambda < 0.4$ and a more accurate result from $M = 70$ for $|J|/\lambda = 0.4 \sim 0.6$ and $M = 80$ for $|J|/\lambda = 0.7$ in the main text.

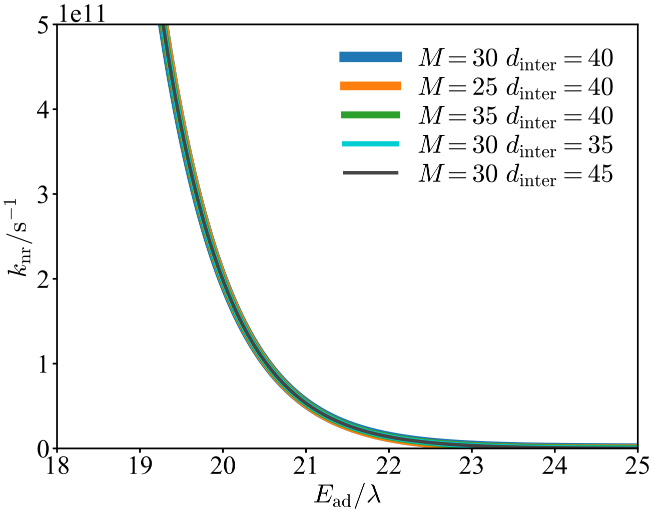

Supplementary Figure 10: Non-radiative decay rate (k_{nr}) spectrum results from different time-dependent density matrix renormalization group (TD-DMRG) computational parameters used for 1D chain lattice with additional inter-molecular vibrations at room temperature.

The excitonic coupling strength is $|J|/\lambda = 1.1$ and the thermal fluctuation of excitonic coupling caused by inter-molecular vibrations is $\Delta J = 0.7\lambda$. The benchmark parameters include the bond dimension M and basis size d_{inter} for the low-frequency inter-molecular vibrations. It can be seen $M = 30$ and $d_{\mathrm{inter}} = 40$ used for simulations in the main text are both enough to obtain converged results.(E_{ad} is the adiabatic excitation energy for each monomer)

Supplementary Note 3. Ab-initio quantum chemistry parameters for azulene

Ab-initio parameters are calculated from density functional theory (DFT) and time-dependent density functional theory (TD-DFT) using B3LYP functional on azulene monomer in the gas phase. The basis used is 6-31G(d). The Gaussian 16[1] program is used for (TD-)DFT calculations. Electron-phonon coupling analysis is performed using the program MOMAP.[2] We evaluated the non-radiative decay from excited state S_1 to ground state S_0. The ab-initio calculated adiabatic excitation energy of azulene monomer is $E_{\mathrm{ad}}^{\mathrm{ab-initio}} = 2.04\,\mathrm{eV}$. We have also evaluated the results of a larger basis set and other functionals for this job. B3LYP/6-311+G(d,p) gives $E_{\mathrm{ad}}^{\mathrm{ab-initio}} = 2.01\,\mathrm{eV}$; M062X/6-31G(d) gives $E_{\mathrm{ad}}^{\mathrm{ab-initio}} = 2.07\,\mathrm{eV}$ and ωb97xd/6-31G(d) gives $E_{\mathrm{ad}}^{\mathrm{ab-initio}} = 2.11\,\mathrm{eV}$. Although slightly different $E_{\mathrm{ad}}^{\mathrm{ab-initio}}$ are given by different functionals and basis sets, we believe ab-initio data from B3LYP/6-31G(d) is already accurate enough for us to use azulene as a platform to qualitatively evaluate the influence of excitonic coupling in real molecules aggregates. In the tables below we display the harmonic frequency for normal modes of the electronic ground state ω_{gs} and harmonic frequency for normal modes of the electronic excited state ω_{ex}. The displacement $\Delta q = q_{\mathrm{ex}} - q_{\mathrm{gs}}$ is used to calculate the electron-phonon coupling as $g = \sqrt{\omega/2}\Delta q$ and the monomer reorganization energy as $\lambda = \frac{\omega_{\mathrm{ex}}^2(\Delta q)^2}{2}$ for each mode. $\langle\psi_{\mathrm{gs}}|\frac{\partial}{\partial q_{\mathrm{gs}}}|\psi_{\mathrm{ex}}\rangle$ is used to calculated the nonadiabatic coupling constant $V = -i\hbar\langle\psi_{\mathrm{gs}}|\frac{\partial}{\partial q_{\mathrm{gs}}}|\psi_{\mathrm{ex}}\rangle$ for each mode. The containing of Supplementary Table. 1 and Supplementary Table. 2 and the coordinate data for optimized structures of studied azulene molecule at ground state S_0 and excited state S_1 have been provided in the source data files in Zenodo with DOI 10.5281/zenodo.8042117.

Supplementary Table 1: Ab-initio quantum chemistry parameters for azulene in the gas phase. (Mode 1 $\sim$ 35)

Mode	$\omega_{gs}/\mathrm{cm}^{-1}$	$\omega_{ex}/\mathrm{cm}^{-1}$	Δq/a.u.	$\langle\psi_{gs}\vert\frac{\partial}{\partial q_{gs}}\vert\psi_{ex}\rangle$/a.u.
1	166.42	194.23	-0.00000	-0.0000000000
2	172.26	116.70	0.00000	0.0000000000
3	322.99	284.09	0.00000	-0.0000000000
4	337.53	334.72	0.28669	-0.0018849253
5	412.94	391.18	10.29530	0.0000067747
6	431.20	405.59	0.00000	0.0000000000
7	499.02	505.09	0.00282	0.0003217778
8	575.15	571.00	0.00000	0.0000000000
9	610.61	501.24	-0.00000	0.0000000000
10	681.98	673.40	-10.32214	-0.0000022405
11	732.07	695.89	0.00000	-0.0000000000
12	746.14	681.54	0.00000	0.0000000000
13	750.19	762.68	0.03634	0.0055018523
14	786.01	724.65	-0.00000	-0.0000000000
15	797.19	761.80	0.00000	0.0000000000
16	831.15	875.51	-22.43270	-0.0000078493
17	882.52	854.80	-0.00000	-0.0000000000
18	915.61	919.38	7.02591	0.0000049750
19	936.28	861.60	-0.00000	0.0000000000
20	969.32	946.44	0.98988	-0.0000014395
21	979.61	798.00	-0.00000	-0.0000000000
22	996.00	988.06	-0.00000	0.0000000000
23	1006.48	973.02	0.00000	-0.0000000000
24	1040.67	1095.49	-0.08285	-0.0053689423
25	1071.98	1050.51	0.00248	0.0011885359
26	1092.04	1104.43	-1.47536	0.0000235202
27	1197.77	1177.73	-0.03371	0.0045815294
28	1253.52	1712.58	-0.60854	0.0267344309
29	1254.03	1265.16	3.12657	-0.0000170047
30	1314.26	1235.88	9.91768	-0.0000147232
31	1333.94	1287.86	-0.19349	-0.0028332459
32	1346.97	1248.90	-0.01002	-0.0036128877
33	1435.84	1503.17	16.08571	0.0000488399
34	1445.04	1371.31	0.18297	-0.0023424613
35	1505.53	1477.47	-0.60990	-0.0002691726

Supplementary Table 2: Ab-initio quantum chemistry parameters for azulene in the gas phase. (Mode 36 $\sim$ 48)

Mode	$\omega_{\mathrm{gs}}/\mathrm{cm}^{-1}$	$\omega_{\mathrm{ex}}/\mathrm{cm}^{-1}$	Δq/a.u.	$\langle\psi_{\mathrm{gs}}\vert\frac{\partial}{\partial q_{\mathrm{gs}}}\vert\psi_{\mathrm{ex}}\rangle$/a.u.
36	1506.63	1442.63	-6.06906	-0.0000155760
37	1551.89	1411.92	0.06602	0.0004269577
38	1598.60	1610.25	0.31767	0.0000029230
39	1649.72	1624.50	9.00449	0.0000580900
40	1661.16	1552.29	-0.13719	-0.0077738223
41	3150.54	3149.55	0.41288	-0.0000065279
42	3152.10	3182.76	-0.00406	0.0004626271
43	3160.30	3181.34	-0.10900	0.0000963708
44	3180.19	3152.67	0.00030	-0.0004665467
45	3189.09	3195.13	0.01485	-0.0000008064
46	3217.50	3226.39	0.17405	0.0002373203
47	3234.83	3231.02	0.00110	0.0011195823
48	3243.99	3260.80	-0.16004	-0.0000125272

Supplementary Note 4. Connections to experiments

As introduced in the main text, we also connect our theoretical findings to experimental synthesized molecular aggregates. We carry out tests in a series of squaraine (SQA) J-aggregate homodimers (dSQA) with varying bridge units to control excitonic coupling between monomers. The excitonic coupling strength $|J|$ and fluorescence quantum yield Φ_{fl} of these dSQAs are experimentally measured in previous studies.[3] Here we calculated their monomer reorganization energy λ with DFT and TD-DFT (B3LYP/6-31G(d) level) to obtain the ratio $|J|/\lambda$. The calculated reorganization energy of monomer SQA is $\lambda = 1666\text{cm}^{-1}$. The measured $|J|$ for SQA-X (X=1∼5) dimer are [830cm^{-1},760cm^{-1},340cm^{-1},260cm^{-1},90cm^{-1}]. The measured Φ_{fl} for SQA-X (X=1∼5) dimer are [0.80,0.78,0.74,0.77,0.71]. In Supplementary FIG. 11 we depict the molecular structure of these dSQAs and compare the calculated $|J|/\lambda$ and measured Φ_{fl} across the series. We observe that the dSQA with the highest Φ_{fl} has $|J|/\lambda \approx 0.5$, which is close to the optimal exciton coupling strength suggested by our theoretical studies. (The coordinate data for optimized structures of studied SQA molecule at ground state S_0 and excited state S_1 has been provided in the source data files in Zenodo with DOI 10.5281/zenodo.8042117.)

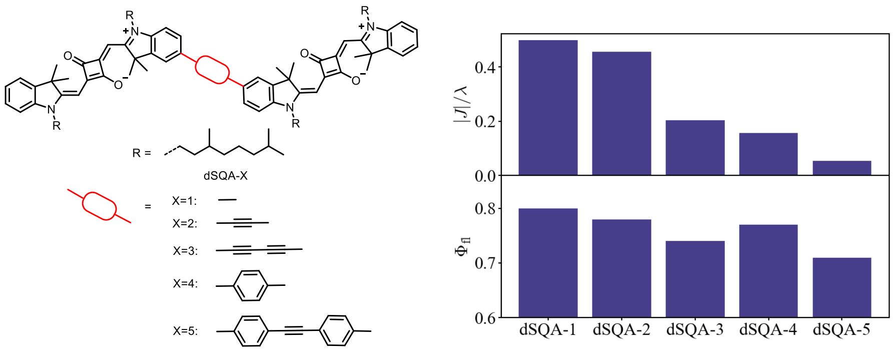

Supplementary Figure 11: The Molecular structure and photophyscical data of SQA and its homodimer.

On the left is the molecule structures of experiment synthesized dye SQA and its homodimer with various bridge units. On the right is the comparison on their photophysical properties.

Supplementary References

(1) Frisch, M. J., Trucks, G. W., Schlegel, H. B., Scuseria, G. E., Robb, M. A., Cheeseman, J. R., Scalmani, G., Barone, V., Petersson, G. A., Nakatsuji, H., Li, X., Caricato, M., Marenich, A. V., Bloino, J., Janesko, B. G., Gomperts, R., Mennucci, B., Hratchian, H. P., Ortiz, J. V., Izmaylov, A. F., Sonnenberg, J. L., Williams-Young, D., Ding, F., Lipparini, F., Egidi, F., Goings, J., Peng, B., Petrone, A., Henderson, T., Ranasinghe, D., Zakrzewski, V. G., Gao, J., Rega, N., Zheng, G., Liang, W., Hada, M., Ehara, M., Toyota, K., Fukuda, R., Hasegawa, J., Ishida, M., Nakajima, T., Honda, Y., Kitao, O., Nakai, H., Vreven, T., Throssell, K., Montgomery, Jr., J. A., Peralta, J. E., Ogliaro, F., Bearpark, M. J., Heyd, J. J., Brothers, E. N., Kudin, K. N., Staroverov, V. N., Keith, T. A., Kobayashi, R., Normand, J., Raghavachari, K., Rendell, A. P., Burant, J. C., Iyengar, S. S., Tomasi, J., Cossi, M., Millam, J. M., Klene, M., Adamo, C., Cammi, R., Ochterski, J. W., Martin, R. L., Morokuma, K., Farkas, O., Foresman, J. B., & Fox, D. J. Gaussian˜16 Revision C.01. Gaussian Inc. Wallingford CT, 2016.

(2) Niu, Y., Li, W., Peng, Q., Geng, H., Yi, Y., Wang, L., Nan, G., Wang, D., & Shuai, Z. Molecular materials property prediction package (momap) 1.0: a software package for predicting the luminescent properties and mobility of organic functional materials, *Mol. Phys.* **116**, 1078–1090 (2018).

(3) Michail, E., Schreck, M. H., Holzapfel, M., & Lambert, C. Exciton coupling effects on the two-photon absorption of squaraine homodimers with varying bridge units, *Phys. Chem. Chem. Phys.* **22**, 18340–18350 (2020).
